# Archaeal Viruses Multiply: Temporal Screening in a Solar Saltern

**DOI:** 10.3390/v7041902

**Published:** 2015-04-10

**Authors:** Nina S. Atanasova, Tatiana A. Demina, Andrius Buivydas, Dennis H. Bamford, Hanna M. Oksanen

**Affiliations:** Department of Biosciences and Institute of Biotechnology, University of Helsinki, Viikinkaari 5, 00014 Helsinki, Finland; E-Mails: nina.atanasova@helsinki.fi (N.S.A.); tatiana.demina@helsinki.fi (T.A.D.); dennis.bamford@helsinki.fi (D.H.B.)

**Keywords:** halovirus, halophilic, archaea, hypersaline, *Halorubrum*, head-tail virus, virus-host interaction, virus morphotype

## Abstract

Hypersaline environments around the world are dominated by archaea and their viruses. To date, very little is known about these viruses and their interaction with the host strains when compared to bacterial and eukaryotic viruses. We performed the first culture-dependent temporal screening of haloarchaeal viruses and their hosts in the saltern of Samut Sakhon, Thailand, during two subsequent years (2009, 2010). Altogether we obtained 36 haloarchaeal virus isolates and 36 archaeal strains, significantly increasing the number of known archaeal virus isolates. Interestingly, the morphological distribution of our temporal isolates (head-tailed, pleomorphic, and icosahedral membrane-containing viruses) was similar to the outcome of our previous spatial survey supporting the observations of a global resemblance of halophilic microorganisms and their viruses. Myoviruses represented the most abundant virus morphotype with strikingly broad host ranges. The other viral morphotypes (siphoviruses, as well as pleomorphic and icosahedral internal membrane-containing viruses) were more host-specific. We also identified a group of *Halorubrum* strains highly susceptible to numerous different viruses (up to 26). This high virus sensitivity, the abundance of broad host range viruses, and the maintenance of infectivity over a period of one year suggest constant interplay of halophilic microorganisms and their viruses within an extreme environment.

## 1. Introduction

During the last couple of decades, archaeal viruses have become a fascinating field of research due to several unique discoveries considering virus morphology, genomics, life cycles, and virus-host interactions [1,2,3,4,5,6,7]. However, these observations are based on only approximately 100 described archaeal viruses and a few culture-independent studies on environmental viral metagenomics. This denotes that our knowledge about viruses infecting archaea lags far behind when compared to what we know about viruses of bacteria and eukaryotes.

All the known archaeal viruses infect extremophiles belonging to either *Euryarchaeota* or *Crenarchaeota* phyla [5]. All these viruses have DNA genomes, and no RNA viruses infecting archaea have yet been isolated [5,6]. Viruses of crenarchaea are famous for their unique morphotypes including lemon-, droplet-, and bottle-shaped, as well as helical and bacilliform ones. However, no crenarchaeal head-tailed viruses have been isolated [1,5]. The majority of the known euryarchaeal viruses, on the other hand, are head-tailed infecting halophilic archaea from the family *Halobacteriaceae* [6]. The other known euryarchaeal virus morphotypes are the icosahedral internal membrane-containing, pleomorphic, and lemon-shaped ones [8,9,10]. Haloarchaea and their viruses constitute the dominant microbial flora of hypersaline environments around the world. Values as high as 10^9^ virus-like_particles per milliliter have been reported for viruses in hypersaline waters [11,12,13]. According to transmission electron microscopy (TEM) analyses, lemon-shaped viruses are considered as the dominant virus morphotype in such environments, although to date, only one such halovirus, His1, has been isolated [9,14,15].

Comparison of viruses has had a jump forward when more virion structures are becoming available. The enormous number of viruses in the virosphere seems to fall into a rather small number of structure-based lineages. Viruses within a lineage may or may not have detectable sequence similarity but they share the common virion architecture and major capsid protein (MCP) fold. This would suggest that the number of individual virus morphotypes is limited due to restricted protein fold space [16,17,18,19,20,21]. Recently, it was shown that an archaeal head-tailed virus has the same MCP fold than tailed bacteriophages (order *Caudovirales*) and eukaryotic herpes viruses, indicating their common ancestry [16]. The icosahedral internal membrane-containing viruses of archaea, bacteria, and eukaryotes represent another distinct group of viruses that are suggested to have evolved from a common ancestor based on their MCP fold and virion architecture [17,18,19,20,21]. These findings offer the first structural insights into deeper analyses about haloarchaeal viruses and their relatedness to other virus groups.

To date, in the advent of metagenomics and bioinformatics with high-throughput sequencing and data handling methods, culturing of microorganisms and their viruses is largely neglected. However, it is still essentially the only means to obtaining reliable structural (up to high resolution), functional, and molecular information about viruses. Giving the example of virus evolution, structural information extends much further than genomic information when searching similarity [18,20] and references therein.

Isolation of viruses is also important for studying their specific interactions with the host organisms. In 2012, we performed the first large-scale spatial screening of haloviruses and their hosts introducing “the global network” of virus-host interactions spanning nine hypersaline environments located on different parts of the world [22]. The obtained 45 new haloarchaeal viruses included a new group of viruses with pleomorphic virions, as well as the first podovirus infecting archaea [16,23,24]. Prior to this screening, the number of known haloarchaeal viruses was around 15 [2,25]. The new virus isolates were shown to infect hosts originating from spatially distant environments indicating that related viruses and hosts are globally distributed. These observations have also been supported by culture-independent studies of haloviruses [4].

If hundreds of virus-host interactions can be detected between spatially distant extreme environments, how would this relate to virus-host dynamics within one environment during different years? To date, temporal screenings of halophilic microorganisms in hypersaline environments have only been performed by culture-independent analyses [3,4,26,27]. When viral populations were monitored for three years by tracking assembled genomes in the Australian hypersaline Lake Tyrrell, it was concluded that at the population level, haloviruses are generally stable for days but dynamic for months to years [3,28]. Species diversity is considered to be generally low at salinities close to saturation, but strain-level diversity (commonly referred to as microdiversity) can be high [29,30]. Viruses are considered as the main force affecting microdiversity by attacking the most dominant strains [27].

In order to increase the number of known archaeal virus isolates and to study virus-host interactions in one hypersaline environment over time, we performed a temporal culture-dependent study of archaeal haloviruses and their hosts in the solar saltern of Samut Sakhon, Thailand, during two consecutive years, 2009 (Samut Sakhon II (SSII)) and 2010 (Samut Sakhon III (SSIII)). We isolated and purified 36 virus isolates, characterized their virions, and compared the virion morphotype distribution to the one obtained from the same environment during the spatial study (Samut Sakhon (SSI)) [22]. Furthermore, it is intriguing to learn how many viral lineages are populated by the virus morphotypes obtained here. We also show that haloarchaeal viruses are dynamic over time and able to infect the hosts isolated a year later. Broad virus host ranges and, conversely, sensitivity of the hosts to a large number of different viruses, seem to be characteristic to archaeal myoviruses and certain *Halorubrum* strains. However, other virus morphotypes, such as the pleomorphic viruses, are more specific to their hosts.

## 2. Materials and Methods

### 2.1. Sample Collection

The samples were collected from a solar saltern in Samut Sakhon, Thailand, 13°32'N; 100°17'E, in November 2009 (sample SSII) and December 2010 (sample SSIII). Samples included salt water and crystals from saltern fields (Supplementary Table S1). The densities of liquid samples were determined by weighing 100 µL aliquots.

### 2.2. Growth Conditions

All cells and viruses were grown aerobically at 37 °C in modified growth medium (MGM) [31,32]. The artificial 30% salt water (SW) (240 g NaCl, 30 g MgCl_2_ × 6H_2_O, 35 g MgSO_4_ × 7H_2_O, 7 g KCl, 5 mL of 1 M CaCl_2_ × 2H_2_O and 80 mL of 1 M Tris-HCl (pH 7.2) per liter) was diluted to obtain 18, 20, or 23% SW in the top-agar layer, solid, and broth media, respectively. MGM also contained 5 g of peptone (Oxoid) and 1 g of Bacto yeast extract (Becton, Dickinson and Company, Sparks, MD, USA) per liter. Fourteen (for solid) and 4 g (for top-agar layer) of agar (Yliopiston Apteekki, Helsinki, Finland) or Bacto agar (Becton, Dickinson and Company) were added per liter of media.

### 2.3. Isolation of Prokaryotes and Their Taxonomic Definition

All prokaryotic strains used in this study are presented in Table 1 (archaea) and Supplementary S2 (bacteria). For strain isolation, salt crystals (3 g) were dissolved in 7 mL of 18% SW, incubated at 37 °C with aeration for three hours. Large impurities were removed by centrifugation (Heraeus Biofuge, 3300 *g*, 3 min, 22 °C), and the supernatants (100 µL) were plated. The liquid samples were plated directly. All the plates were incubated for up to 21 days. The obtained colonies were colony-purified three consecutive times, and the whole cell protein patterns of pure cultures were analyzed by sodium dodecyl sulfate 16% polyacrylamide gel electrophoresis (SDS-PAGE) [33].

**Table 1:** Archaeal strains used in this study.

Sample ^a^	Nbr	Strain	16S rRNA Gene Partial Sequence GenBank Acc. No. and Length (bp)	Reference
SSII	1	*Halorubrum* sp. SS6-1	KJ917631 (1315)	This study
	2	*Halorubrum* sp. SS6-2	KJ917632 (1343)	This study
	3	*Halolamina* sp. SS6-3	KJ917633 (996)	This study
	4	*Halobacterium* sp. SS6-4	KJ917634 (1343)	This study
	5	*Halobacterium* sp. SS6-5	KJ917635 (1354)	This study
	6	*Halobellus* sp. SS6-7	KJ917636 (1331)	This study
	7	*Haloarcula* sp. SS7-2	KJ917637 (1345)	This study
	8	*Halorubrum* sp. SS7-4	JN971009 (1333)	[23]
	9	*Haloarcula* sp. SS8-1	KJ917638 (1342)	This study
	10	*Halorubrum* sp. SS8-2	KJ917639 (1338)	This study
	11	*Haloarcula* sp. SS8-3	KJ917640 (1341)	This study
	12	*Haloarcula* sp. SS8-4	KJ917641 (1335)	This study
	13	*Haloarcula* sp. SS8-5	KJ917642 (1357)	This study
	14	*Halorubrum* sp. SS8-7	KJ917643 (1292)	This study
	15	*Haloterrigena* sp. SS9-2	KJ917644 (1323)	This study
	16	*Halogeometricum* sp. SS9-3	KJ917645 (1323)	This study
	17	*Halogranum* sp. SS9-5	KJ917646 (1328)	This study
	18	*Haloferax* sp. SS9-6	KJ917647 (1326)	This study
	19	*Halorubrum* sp. SS9-12	KJ917648 (1345)	This study
SSIII	20	*Halorubrum* sp. SS10-3	KJ917649 (1345)	This study
	21	*Haloarcula* sp. SS10-4	KJ917650 (1317)	This study
	22	*Natrinema* sp. SS10-5	KJ917651 (1348)	This study
	23	*Haloferax* sp. SS10-6	KJ917652 (1338)	This study
	24	*Haloferax* sp. SS10-7	KJ917653 (1334)	This study
	25	*Halorubrum* sp. SS10-9	KJ917654 (1342)	This study
	26	*Halogranum* sp. SS11-3	KJ917655 (1342)	This study
	27	*Halogranum* sp. SS13-4	KJ917656 (1352)	This study
	28	*Halogranum* sp. SS13-5	KJ917657 (1353)	This study
	29	*Halogranum* sp. SS13-6	KJ917658 (1329)	This study
	30	*Haloterrigena* sp. SS13-7	KJ917659 (1344)	This study
	31	*Haloterrigena* sp. SS13-8	KJ917660 (1343)	This study
	32	*Haloterrigena* sp. SS13-10	KJ917661 (1348)	This study
	33	*Halogranum* sp. SS13-11	KJ917662 (1349)	This study
	34	*Halorubrum* sp. SS13-12	KJ917663 (1321)	This study
	35	*Halorubrum* sp. SS13-13	KJ917664 (1306)	This study
	36	*Haloarcula* sp. SS13-14	KJ917665 (1336)	This study
CC	37	*Halorubrum* sp. SS1-3	JN196470 (1330)	[22]
	38	*Halorubrum* sp. SS5-4	JN196482 (1401)	[22]
	39	*Halorubrum* sp. SP3-3	JN196487 (1414)	[22]
	40	*Haloarcula hispanica* ATCC 33960	U68541	[34]
	41	“Haloarcula californiae” ATCC 33799	AB477984	[35]
	42	*Haloarcula japonica* TR1 ATCC 49778	NR_028234	[36]
	43	*Haloarcula marismortui* ATCC 43049	X61688	[37,38]
	44	*Haloarcula quadrata* ATCC 700850	AB010964	[39]
	45	“Haloarcula sinaiiensis” ATCC 33800	D14129	[35]
	46	*Haloarcula vallismortis* ATCC 29715	AB355982	[40,41]
	47	*Halorubrum sodomense* DSM 33755	D13379	[42]

**a.** SSII, Samut Sakhon sample 2009; SSIII, Samut Sakhon sample 2010; CC, culture collection strains.

For partial 16S rRNA gene sequencing, PCR was performed as described previously [22]. The PCR products were sequenced using either archaeal primers D30 and D56 [43] or bacterial pA, or pHr primers [44] at BGI Tech Solutions Co., Ltd, and at the Institute of Clinical-Theoretical Medicine Sequencing Unit (University of Helsinki). Bacterial strains were sequenced only in one direction with either pA or pHr primer. Geneious version 6.1.6 software created by Biomatters (available from http://www.geneious.com/) was used for sequence assembly, and assembled sequences were analyzed using BLASTN [45] and classified at the genus level using a threshold of 95% identity. The phylogenetic tree of archaeal isolates and reference strains was constructed using maximum likelihood method and 1000 bootstraps values with the Molecular Evolutionary Genetics Analysis (MEGA) version 5.05 software [46]. The sequence data have been deposited in GenBank database (Table 1 and Supplementary S2).

### 2.4. Isolation of Viruses and Their Characterization

All viruses used in this study are presented in Table 2 and Supplementary S3. For virus isolation, water samples or salt crystals (3 g) dissolved in 7 mL of 18% or 6% SW, were centrifuged (Heraeus Biofuge, 15,700 *g*, 5 min, 22 °C), and 100 µL of the supernatant was mixed with 300 µL of cells at exponential or early stationary phase. Three ml of molten top agar (50–60 °C) were added and the mixture was plated on MGM-plates. After 2–5 days of incubation, plaques were picked and plaque-purified three consequent times. Virus stocks were prepared from confluent or semi-confluent plates. For virus purification, virus particles were precipitated from the stocks with polyethylene glycol 6000, subjected to rate-zonal sucrose gradient centrifugation and when appropriate to CsCl isopycnic density gradient centrifugation using 18% SW as a buffer, as described previously [22].

Proteins of purified virions were analyzed by tricine-SDS-PAGE (14% acrylamide in the separation gel) [47]. Protein concentrations were determined by the Bradford assay using bovine serum albumin as a standard [48]. Chloroform sensitivity of the viruses was tested by incubating the virus stock with chloroform (20% (v/v) final concentration) for 15 min at 22 °C. After incubation, the number of infective particles was determined by the plaque assay.

For transmission electron microscopy, purified viruses were negatively stained with 1% (w/v) potassium phosphotungstate (pH 6.5) for 5 s or 3% (w/v) uranyl acetate (pH 4.5) for 30 s. The micrographs were taken with JEOL 1200 EX or JEOL 1400 electron microscopes operating at 80 kV (Electron Microscopy Unit, Institute of Biotechnology, University of Helsinki).

### 2.5. Virus-Host Interactions Test

Sensitivity of all isolated strains (except *Halogranum* sp. SS13-5 and *Halorubrum* sp. SS13-13) and culture collection strains against all virus isolates was determined by a spot-on-lawn test. Drops (10 µL) of undiluted and 1:100 diluted virus stocks were placed on the lawn that was prepared by mixing the early stationary growing strain (300 µL) and soft agar (3 mL). After 3–5 days of incubation plates were analyzed for the presence of growth inhibition. All positive results were verified by the plaque assay.

**Table 2:** Viruses used in this study.

Sample^a^		Virus Isolate No.	Virus	Nomenclature	Plaque Morphology	Stock Titer (pfu ml^−1^)	Chloroform Sensitivity	Morphotype	Isolation Host	Reference for the Virus
SSII	SS9	1	HRTV-13	*Halorubrum* tailed virus 13	Clear	3.6 × 10^9^	NS	Myovirus	*Halorubrum* sp. SS8-2	This study
	SS9	2	HRTV-14	*Halorubrum* tailed virus 14	Clear	3.0 × 10^10^	NS	Myovirus	*Halorubrum* sp. SS6-2	This study
	SS6	3	HRTV-15	*Halorubrum* tailed virus 15	Clear	1.4 × 10^10^	NS	Myovirus	*Halorubrum* sp. SS6-2	This study
	SS7	4	HRTV-16	*Halorubrum* tailed virus 16	Clear	4.9 × 10^10^	NS	Myovirus	*Halorubrum* sp. SS6-2	This study
	SS6	5	HRTV-17	*Halorubrum* tailed virus 17	Clear	1.0 × 10^9^	NS	Myovirus	*Halorubrum* sp. SS9-12	This study
SSIII	SS10	6	HSTV-4	*Halorubrum sodomense* tailed virus 4	Clear	4.5 × 10^7^	NS	Myovirus	*Halorubrum sodomense*	This study
	SS10	7	HRTV-18	*Halorubrum* tailed virus 18	Clear	1.4 × 10^9^	NS	Myovirus	*Halorubrum* sp. SS10-3	This study
	SS13	8	HRTV-19	*Halorubrum* tailed virus 19	Clear	2.7 × 10^8^	NS	Myovirus	*Halorubrum* sp. SS10-3	This study
	SS10	9	HRTV-20	*Halorubrum* tailed virus 20	Turbid	5.5 × 10^9^	NS	Myovirus	*Halorubrum* sp. SS10-9	This study
	SS10	10	HRTV-21	*Halorubrum* tailed virus 21	Turbid	1.4 × 10^7^	NS	Myovirus	*Halorubrum* sp. SS10-9	This study
	SS10	11	HRTV-22	*Halorubrum* tailed virus 22	Clear	5.7 × 10^9^	NS	Myovirus	*Halorubrum* sp. SS10-9	This study
	SS13	12	HRTV-23	*Halorubrum* tailed virus 23	Turbid	5.8 × 10^9^	NS	Myovirus	*Halorubrum* sp. SS10-9	This study
	SS13	13	HRTV-24	*Halorubrum* tailed virus 24	Turbid	2.6 × 10^8^	NS	Myovirus	*Halorubrum* sp. SS10-9	This study
	SS10	14	HRTV-25	*Halorubrum* tailed virus 25	Turbid	1.2 × 10^11^	NS	Myovirus	*Halorubrum* sp. SS13-12	This study
	SS10	15	HCTV-6	“Haloarcula californiae” tailed virus 6	Clear	1.3 × 10^9^	NS	Myovirus	“Haloarcula californiae”	This study
	SS10	16	HCTV-7	“Haloarcula californiae” tailed virus 7	Turbid	1.3 × 10^10^	NS	Myovirus	“Haloarcula californiae”	This study
	SS10	17	HCTV-8	“Haloarcula californiae” tailed virus 8	Turbid	5.0 × 10^10^	NS	Myovirus	“Haloarcula californiae”	This study
	SS10	18	HCTV-9	“Haloarcula californiae” tailed virus 9	Turbid	2.6 × 10^10^	NS	Myovirus	“Haloarcula californiae”	This study
	SS10	19	HCTV-10	“Haloarcula californiae” tailed virus 10	Turbid	1.5 × 10^10^	NS	Myovirus	“Haloarcula californiae”	This study
	SS10	20	HCTV-11	“Haloarcula californiae” tailed virus 11	Turbid	4.3 × 10^10^	NS	Myovirus	“Haloarcula californiae”	This study
	SS11	21	HCTV-12	“Haloarcula californiae” tailed virus 12	Turbid	8.0 × 10^10^	NS	Myovirus	“Haloarcula californiae”	This study
	SS12	22	HCTV-13	“Haloarcula californiae” tailed virus 13	Clear	3.0 × 10^9^	NS	Myovirus	“Haloarcula californiae”	This study
	SS12	23	HCTV-14	“Haloarcula californiae” tailed virus 14	Turbid	4.5 × 10^10^	NS	Myovirus	“Haloarcula californiae”	This study
	SS12	24	HCTV-15	“Haloarcula californiae” tailed virus 15	Clear	7.0 × 10^10^	NS	Myovirus	“Haloarcula californiae”	This study
	SS10	25	HJTV-3	*Haloarcula japonica* tailed virus 3	Clear	7.1 × 10^8^	NS	Myovirus	*Haloarcula japonica*	This study
	SS10	26	HRTV-26	*Halorubrum* tailed virus 26	Turbid	1.7 × 10^8^	NS	Myovirus	*Halorubrum* sp. SS13-13	This study
	SS13	27	HRTV-27	*Halorubrum* tailed virus 27	Clear	5.0 × 10^6^	NS	Myovirus	*Halorubrum* sp. SS13-13	This study
SSII	SS6	28	HRTV-28	*Halorubrum* tailed virus 28	Turbid	6.0 × 10^10^	NS	Siphovirus	*Halorubrum* sp. SS8-7	This study
	SS7	29	HRTV-29	*Halorubrum* tailed virus 29	Turbid	4.5 × 10^12^	NS	Siphovirus	*Halorubrum* sp. SS7-4	This study
	SS7	30	HATV-3	*Haloarcula* tailed virus 3	Turbid	1.0 × 10^11^	NS	Siphovirus	*Haloarcula* sp. SS8-5	This study
SSIII	SS10	31	HCTV-16	“Haloarcula californiae” tailed virus 25	Turbid	1.5 × 10^11^	NS	Siphovirus	“Haloarcula californiae”	This study
SSII	SS8	32	HRPV-6	*Halorubrum* pleomorphic virus 6	Turbid	1.1 × 10^12^	NS	Pleomorphic	*Halorubrum* sp. SS7-4	[23]
	SS6	33	HRPV-7	*Halorubrum* pleomorphic virus 7	Turbid	8.7 × 10^10^	−1 log	Pleomorphic	*Halorubrum* sp. SS5-4	This study
	SS8	34	HRPV-8	*Halorubrum* pleomorphic virus 8	Turbid	4.9 × 10^9^	NS	Pleomorphic	*Halorubrum* sp. SP3-3	This study
SSIII	SS13	35	HAPV-2	*Haloarcula* pleomorphic virus 2	Turbid	1.6 × 10^9^	−3 log	Pleomorphic	*Haloarcula* sp. SS13-14	This study
SSIII	SS13	36	HCIV-1	“Haloarcula californiae” icosahedral virus 1	Clear	5.8 × 10^10^	−2 log	Icosahedral	“Haloarcula californiae”	This study

**a.** SSII, Samut Sakhon sample 2009; SSIII, Samut Sakhon sample 2010.

## 3. Results

### 3.1. All Isolated Archaeal Strains Belonged to Ten Genera of the Halobacteriaceae Family

We performed a temporal sampling in the solar saltern of Samut Sakhon (Thailand) during two consecutive years (samples SSII, November 2009; samples SSIII, December 2010). The samples included salt crystals (large crystals directly from the field or rinsed finely ground crystals), and salt water (ρ = 1.02–1.15 g ml^−1^, Supplementary Table S1). Salt crystals and liquid samples were collected from the same 10 m^2^ area on the salt field (Supplementary Table S1).

Using modified growth medium (~3.15 M NaCl, ρ = 1.15 g mL^−1^), we first isolated halophilic prokaryotes from SSII and SSIII sample sets. The different strains were selected based on the colony appearance and whole cell protein pattern analysis by SDS-PAGE (data not shown). We classified all the unique isolates at the genus level, using a threshold of 95% 16S rRNA gene sequence identity [49] to representative haloarchaeal species with complete 16S rRNA gene sequence available in GenBank database. The isolates included 36 archaeal (Table 1) and 15 bacterial strains (Supplementary Table S2), of which the archaeal ones were used for virus isolation.

**Figure 1:**
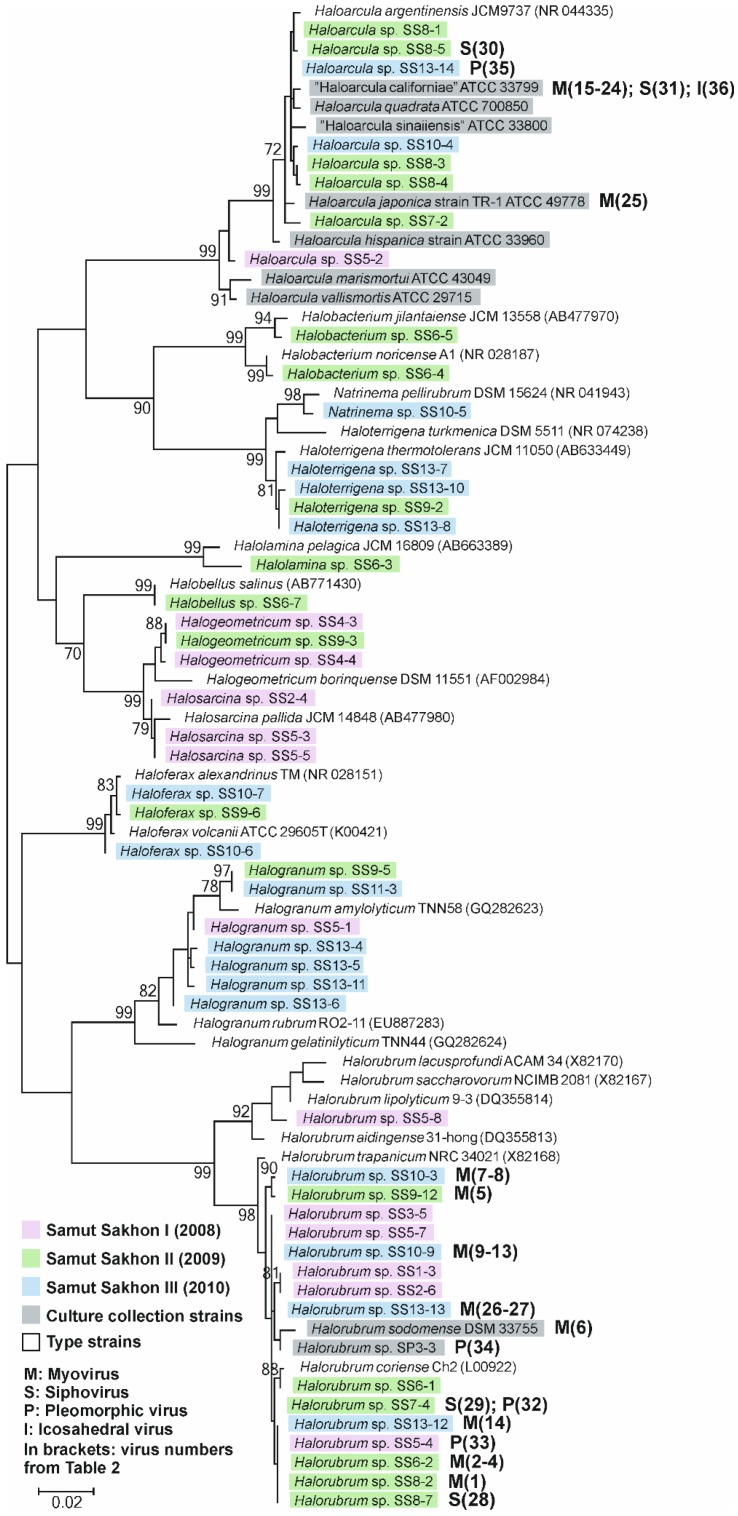
Maximum likelihood phylogenetic tree of haloarchaeal partial 16S rRNA gene sequences. The archaeal strains obtained from SSI samples [22] are marked with pink. SSII and SSIII strains are highlighted with green and blue, respectively. The culture collection strains used in this study are highlighted with grey. The accession numbers of SSII and SSIII strains are listed in Table 1. Accession numbers of SSI strains can be found from [22]. Reference strains have accession numbers but no color codes. Virus isolates are marked with their virus numbers (See Table 2) in brackets and a letter indicating the virus morphotype (See the bottom left corner of the figure) after the strain name. Bar (0.02) represents inferred substitutions per nucleotide substitution.

The archaea included 19 and 17 isolates from SSII and SSIII samples, respectively. The majority of the strains (30 out of 36) were isolated from salt crystals and only six from salt water samples. On the basis of their partial 16S rRNA gene sequences, we constructed a maximum likelihood phylogenetic tree (Figure 1) that also includes the archaeal strains from SSI samples [22], culture collection strains (Table 1) as well as appropriate representative species. Archaea from the samples SSII and SSIII belonged to ten genera of the *Halobacteriaceae* family: *Halorubrum* (10 strains), *Haloarcula* (seven strains), *Halogranum* (six strains), *Haloterrigena* (four strains), *Haloferax* (three strains), *Halobacterium* (two strains), *Halogeometricum* (one strain), *Halobellus* (one strain), *Natrinema* (one strain), and *Halolamina* (one strain). Four genera, *Halogranum*, *Halorubrum*, *Haloferax,* and *Haloarcula* included isolates from both SSII and SSIII samples. All *Halobellus* and *Halobacterium* strains were found from SSII samples, while *Haloterrigena* and *Natrinema* strains originated from SSIII samples.

### 3.2. Thirty Six Euryarchaeal Virus Isolates Were Assigned to Known Virus Morphotypes

We used a culture-dependent approach in order to isolate viruses from SSII and SSIII samples on endogenous archaeal strains derived from the same samples (see above). To increase the likelihood of finding new viruses from SSII samples, we included three culture collection strains (*Halorubrum* sp. SS1-3, SS5-4, and SP3-3 [22]) that are known to be susceptible to several haloviruses. Later, we included another set of eight culture collection strains for SSIII samples to enhance the search for new viruses even further. The group included *Haloarcula hispanica*, “Har. californiae”, *Har.* *japonica*, *Har. marismortui*, *Har. quadrata*, “Har. sinaiiensis”, *Har. vallismortis,* and *Halorubrum sodomense*.

**Figure 2:**
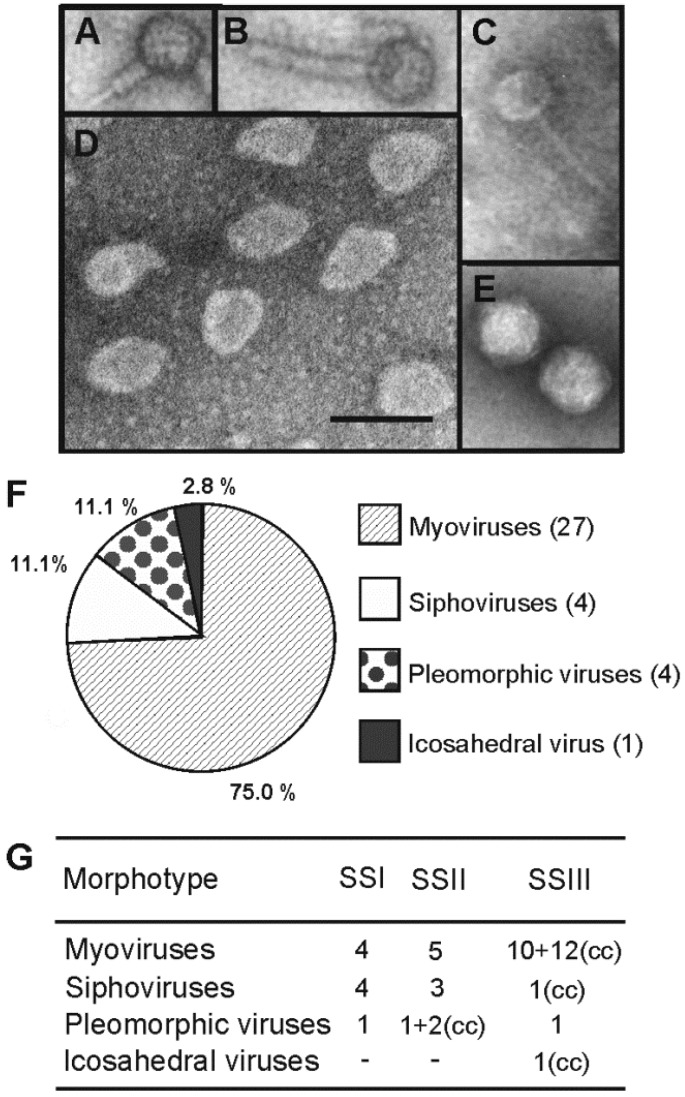
Transmission electron micrographs and morphotype distribution of the virus isolates. (**A**–**B**) Myovirus morphotype (Isolate No. 4, HRTV-16) with the tail in (**A**) contracted and (**B**) extended conformations; (**C**) Siphovirus morphotype (Isolate No. 28, HCTV-28); (**D**) Pleomorphic virus morphotype (Isolate No. 32, HRPV-6); (**E**) Icosahedral virus morphotype (Isolate No. 36, HCIV-1). Scale bar in D is 100 nm for all panels; (**F**) The percentages of different virus morphotypes isolated from SSII and SIII samples; (**G**) Numbers of viruses isolated from samples SSI [22], SSII, and SSIII on the endogenous strains derived from the same sample and culture collection (cc) strains.

With this approach, we obtained 45 archaeal virus isolates in total on either *Halorubrum* or *Haloarcula* strains. Only six of the virus isolates were obtained from salt water samples. The rest were isolated from salt crystals suggesting that terrestrial hypersaline elements are rich in viruses. Most viruses formed plaques after three days of incubation, and the plaque morphologies were either clear (Supplementary Figure S1 A–C) or variably turbid (Supplementary Figure S1 D–I). The diameters of the plaques varied from approximately one to 15 millimeters. We plaque-purified all the virus isolates three consecutive times and prepared virus stocks (see Materials and Methods) with titers ranging from 10^6^ to 10^12^ PFU/mL. Using polyethylene glycol-sodium chloride precipitation, sucrose rate-zonal centrifugation, and CsCl isopycnic density gradient centrifugation (when appropriate), 36 out of 45 virus isolates were successfully purified. The remaining nine isolates could not be purified due to low number of infective particles or instability during purification (Supplementary Table S3). All these viruses with unknown virion morphology were isolated from SSIII/SS10 sample (Supplementary Table S1), and mainly on *Hrr. sodomense* (seven isolates; Supplementary Table S3). Nine out of the 36 purified viruses were isolated on SSII strains and 11 viruses on strains from SSIII (Figure 1 and Figure 2). In addition, the usage of culture collection strains increased the number of obtained isolates by 16. Among SSII and SSIII strains, we found the highest number of viruses (20) on *Halorubrum* strains, and among culture collection strains on “Haloarcula californiae” for which 12 virus isolates were discovered.

The uniqueness of the viral isolates was determined based on the following characteristics: plaque morphology (Supplementary Figure S1; Table 2), virion morphotype determined by TEM (Figure 2; Table 2), structural protein pattern of purified virus particles (Supplementary Figure S2), sensitivity of the virus infectivity to chloroform (Table 2), virus host range (Supplementary Tables S4 and S5), and efficiency of plating (EOP) on different host strains (Supplementary Tables S4 and S5). The 36 purified virus isolates differed from each other based on these criteria and were thus chosen for further studies. The isolates were numbered from 1 to 36 and named according to virion morphotype and the original isolation host organism (Table 2). Virus isolates with unknown morphology were numbered from 37 to 45 (Supplementary Table S3).

Virion morphotypes of the purified viruses (Nos. 1–36) were determined by TEM (Figure 2A–E). The majority (31; 86%) of the viruses were head-tailed, of which 27 and four were myo- and siphoviruses, respectively. No podoviruses were isolated. The icosahedral heads of the myo- and siphoviruses were ~55–85 nm and ~45–75 nm in diameter, respectively. In addition, we identified four pleomorphic viruses, which were ~45–85 nm in diameter when negatively stained with phosphotungstic acid (Figure 2D). These viruses include *Halorubrum* pleomorphic virus 6 (HRPV-6, No. 32), which has previously been described in detail [23,24]. We also found one tailless icosahedral virus, “Haloarcula californiae” icosahedral virus 1 (HCIV-1, No. 36), with a diameter of ~70 nm and most probably with an internal membrane (Figure 2E). Thus, only four different virus morphotypes were observed and distributed among myo- (75.0%), sipho- (11.1%), pleomorphic (11.1%), and icosahedral (2.8%) viruses. This ratio is unexpectedly similar to that of Samut Sakhon 2008 (SSI) samples (Figure 2G), as well as to the total morphotype distribution obtained from the spatial culture-dependent sampling of nine geographically distant hypersaline environments [22]. Among all viruses, only two pleomorphic viruses, HRPV-7 and HAPV-1 (Nos. 33 and 35), and the icosahedral virus, HCIV-1 (No. 36), were sensitive to chloroform which reduced the infectivity by at least one order of magnitude (Table 2).

### 3.3. Multiple Virus-Host Interactions, in which Myoviruses Were the Most Promiscuous, Were Observed

Using a spot-on-lawn test for preliminary screening and subsequent plaque assay to verify the positive results, we determined specific virus-host interactions. All together ~3000 virus-strain pairs were cross-tested: all isolated viruses (45; Table 2 and Supplementary S3) against all archaeal (45; Table 1) and bacterial strains (15; Supplementary Table S2). However, *Halogranum* sp. SS13-5 and *Halorubrum* sp. SS13-13 were not included, because these strains do not form an adequate lawn. No interactions between viruses and bacteria were observed, showing that all the tested viruses are archaea-specific.

**Figure 3:**
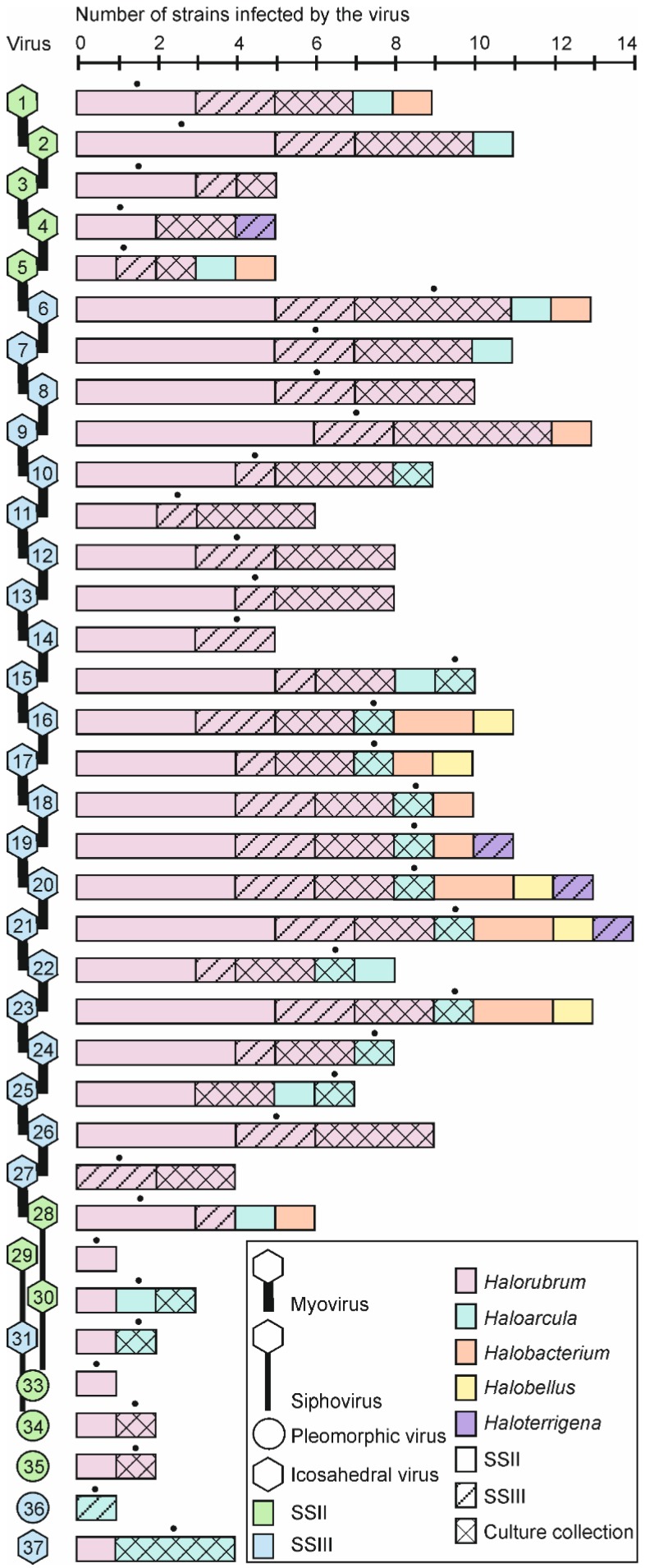
Host range of viruses. Genus distribution of the virus host strains is indicated by a colored column corresponding to the number of infected strains (Supplementary Tables S3 and S4). Black dot on top of the column indicates the genus of the original isolation host of the virus. The color, pattern, and morphotype codes are presented at the bottom of the figure. See Table 2 for virus numbers.

We detected 268 specific virus-host interactions among the 36 virus isolates with known morphotype (Supplementary Tables S4 and S5). By grouping all the interactions on the basis of virus morphotype, we observed that 91.8% of all interactions were those of myoviruses, and only 4.5%, 2.2%, and 1.5% were caused by sipho-, pleomorphic, and icosahedral viruses, respectively (Figure 3; Supplementary Tables S4 and S5). Viruses had on average seven interactions with archaea. The relative number of specific virus-host interactions was also the highest within the myovirus group, where all viruses had on average nine interactions with the archaeal host strains. For sipho- and pleomorphic viruses the corresponding numbers are three and two, respectively. The only icosahedral virus infected four strains.

### 3.4. Broad Host Ranges Covering Archaea from Different Genera Were Characteristic to Many Myovirus Isolates

All myoviruses infected at least three *Halorubrum* strains. HCTV-12 (No. 21 from SSIII) had the broadest host range including 14 different strains from five genera (Figure 3). Eight myoviruses were specific only for *Halorubrum* strains, but the others, 19 in total, infected strains belonging to at least two and up to five different genera. Among these, for the first time, we identified viruses infecting a *Halobellus* strain (Figure 3; myoviruses Nos. 16, 17, 20, 21, and 23). The other myovirus host strains belonged to either *Halorubrum*, *Haloarcula*, *Halobacterium*, or *Haloterrigena* genera.

In general, the EOP of the viruses on different hosts varied up to ten orders of magnitude (Supplementary Tables S3–S5). Typically, myovirus titers were higher on their own isolation host than on the other host strains. However, in some cases, myoviruses could infect other strains more efficiently than their original isolation host having no more than one order of magnitude difference in the EOP (Supplementary Table S4). In all these cases where one order of magnitude higher EOPs were observed on a new host strain, the virus and the strain had been isolated from samples collected during different years, or the host was one of the culture collection strains.

Siphoviruses had narrower host ranges than myoviruses. One out of four siphoviruses infected only the original isolation host (*Haloarcula* strain) (Figure 3). The rest infected two to six strains belonging to two or three genera (*Halorubrum*, *Haloarcula*, and *Halobacterium*). Among siphoviruses, the highest EOP was always on the original host strain (Supplementary Table S4). Pleomorphic viruses were the most specific infecting only one or two strains from one genus (*Halorubrum* or *Haloarcula*) with the same or one order of magnitude lower EOP (Figure 3; Supplementary Table S5). The icosahedral virus, isolated on “Haloarcula californiae”, could also infect *Har. japonica* with the same EOP, and *Halorubrum* sp. SS7-4 and *Har. hispanica* with a significantly lower EOPs (Figure 3; Supplementary Table S5).

### 3.5. Halorubrum Strains Were the Most Susceptible to Viruses

**Figure 4:**
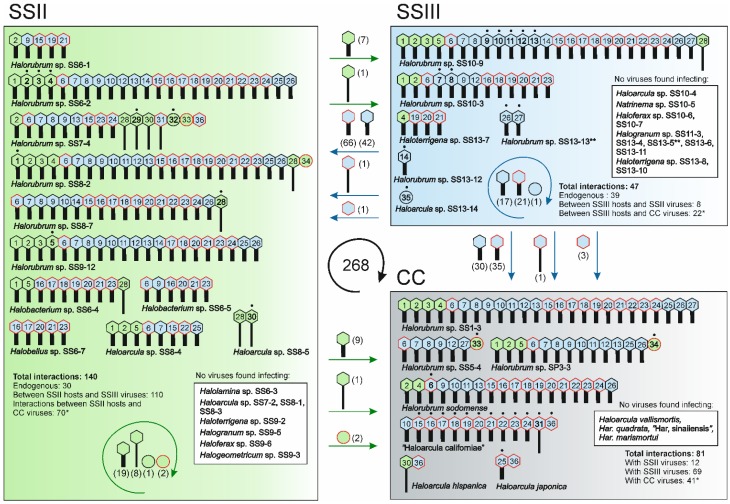
Virus-host interactions within and across the two samples from Samut Sakhon (2009, SSII and 2010, SSIII) and with the culture collection (CC) strains. Large rectangles represent virus sensitivities of the strains isolated from SSII (green) and SSIII (blue), and the culture collection strains (grey). Virus morphotypes and numbers are shown as in Table 2 and Figure 3 and are colored according to the samples from which they originate. Viruses originally isolated on culture collection strains are marked with red. Dots on top of the viruses with bolded numbers indicate that the strain is the original isolation host. Straight arrows (colored as above) indicate interactions between viruses and strains from different samples or between viruses and the culture collection strains. The number of interactions for each virus morphotype is shown in brackets. The green and blue curved arrows represent interactions of viruses and hosts isolated during the same year (endogenous interactions). The numbers of both endogenous and cross-sample interactions are shown for each year. Numbers of interactions with the culture collection strains (marked with an asterisk) are included in the numbers of endogenous and/or cross-sample interactions (See also Tables S4 and S5). The strains that are not infected by viruses are in white boxes. *Halogranum* sp. SS13-5 and *Halorubrum* sp. SS13-13 marked with double asterisks were used in the initial virus screening, but not in the interaction study due to difficulties in obtaining a dense lawn. The black curved arrow in the center shows the total number of all interactions.

Half of our archaeal isolates and the culture collection archaeal strains were hosts for at least one virus. No viruses were found for strains of *Halolamina*, *Halogranum*, *Haloferax*, *Halogeometricum*, or *Natrinema* genera. Although, originally all the viruses were isolated either on *Halorubrum* or *Haloarcula* strains, cross-testing (see above) revealed that also strains from the genera *Halobacterium*, *Halobellus*, and *Haloterrigena* were infected by the isolated myoviruses (Figure 4 and Supplementary S4). However, the majority of the virus interactions were with *Halorubrum* strains (~80%; in total 215 interactions). Other strains had significantly less interactions with viruses: ~10% for *Haloarcula* (in total 28 interactions), ~6% for *Halobacterium* (in total 16 interactions), ~2% for *Halobellus* (in total five interactions) and ~1.5% for *Haloterrigena* strains (in total four interactions) (Figure 3 and Figure 4).

The archaeal virus host strains were infected by up to 26 viruses, but on the average they supported the propagation of 11 viruses. All our ten *Halorubrum* strains were infected by viruses and the average number of interactions per host strain was 15, while the corresponding numbers for *Haloarcula*, *Halobacterium*, *Halobellus*, and *Haloterrigena* strains were five, eight, five, and four, respectively. Apart from *Halorubrum*, *Halobacterium*, and *Halobellus*, the genera *Haloarcula* and *Haloterrigena* also included strains for which no interactions were detected (Figure 4).

We revealed a group of ten *Halorubrum* strains (*Halorubrum sodomense*, *Hrr.* sp. SS1-3, SS9-12, SS8-2, SS6-2, SS10-9, SS7-4, SS8-7, SS10-3, and SP3-3) that were susceptible to numerous viruses (from 13 up to 26). Notably, these strains originated from samples SSII and SSIII or belonged to the culture collection. In addition, *Halobacterium* sp. SS6-4, *Haloarcula* sp. SS8-4, and “Haloarcula californiae” were sensitive to ten, eight, and 13 viruses, respectively. Only *Halorubrum* sp. SS7-4 was infected by viruses representing all four different morphotypes, whereas about half of the virus host strains were sensitive to viruses of only one morphotype (Figure 4). *Halorubrum* sp. SS8-2 and “Haloarcula californiae” were sensitive to viruses representing three different morphotypes and seven hosts from the genera *Halorubrum*, *Haloarcula*, or *Halobacterium* were infected by viruses with two morphotypes (Figure 4). Altogether 12 hosts from the genera *Halorubrum*, *Haloarcula*, *Halobacterium*, *Halobellus*, or *Haloterrigena* were infected only by myoviruses (Figure 4).

### 3.6. Virus-Host Interactions Were Observed Within and Across Samples

The distribution of the total number of 268 interactions among different strains showed that SSII strains were involved in half of the virus-host interactions (140 interactions), whereas SSIII strains had 47 interactions, and culture collection strains 81 interactions (Figure 4). Consequently, the average number of interactions was 13 per SSII virus host strain, eight per SSIII host strain, and 12 per culture collection host strain. Approximately 42% and 65% of the SSII and SSIII archaeal isolates, respectively, did not interact with the available viruses at all (Figure 4). Four out of eleven (~36%) tested culture collection strains were resistant to the viruses obtained here.

We revealed 30 and 39 endogenous virus-host interactions in SSII and SSIII samples, respectively, comprising ~25% of all interactions (Figure 4). The total number of cross-sample interactions between SSII viruses and SSIII strains was eight (7%), but 110 (93%) between SSIII viruses and SSII strains. Excluding interactions with the culture collection strains, almost half of the total interactions (~44%) occurred temporally between SSII (2009) and SSIII (2010) samples. In these interactions, myoviruses isolated from SSIII samples had a major impact having 108 interactions (~40% of all interactions) with SSII archaea that were mainly *Halorubrum* strains (Figure 4).

Culture collection strains had numerous interactions with the virus isolates. There were 12 and 69 interactions with SSII and SSIII viruses, respectively, composing ~5% and ~26% of the total interactions (Figure 4). Viruses originally isolated from SSIII samples on culture collection strains interacted 70 times with SSII strains, 22 times with SSIII strains, and 41 times with culture collection strains, giving a total number of 133 interactions (~50% of all interactions). Pleomorphic viruses isolated on culture collection strains had one more interaction per virus (two sensitive strains) compared to the other viruses of the same morphotype that were specific to their endogenous isolation host strains. All the observed additional interactions were with SSII strains.

To summarize, most of the interactions were those of myoviruses. If only their interactions with *Halorubrum* strains are taken into account, the number of interactions is 202. This comprises ~75% of all interactions, suggesting that, at least according to this culture-dependent study, myoviruses and *Halorubrum* strains are the most dominant entities in the virus-host interaction network of highly saline environments. No strong temporal correlations were observed in the virus-host interactions detected during the two sampling years (Table 3). It seems that SSII hosts were preferred by both endogenous viruses (SSII viruses), viruses isolated a year after (SSIII), and viruses isolated a year after on culture collection hosts (Table 3). A slight increase in interactions was observed for viruses isolated from SSIII samples on culture collection hosts indicating that by isolating the virus on a “foreign” host strain and then introducing it back to the local ones might increase its host range (Table 3). The elevated numbers of interactions were observed for this type of viruses (including all morphotypes) for all host types. The alternative is that the viruses isolated on culture collection hosts were those with the broadest host ranges in the first place.

In addition to the 268 specific virus-host interactions, 73 interactions were observed for the virus isolates with unknown morphotypes (Supplementary Table S3). These viruses (nine in total) had a relatively wide host range (5–10 hosts) and seven out of nine isolates could infect hosts from 2–3 genera (Supplementary Table S3). All these isolates infected strains from both Samut Sakhon samples as well as from the culture collection strain group.

**Table 3:** The number of virus-host interactions according to isolation year, virus morphotype, and isolation host.

Hosts	Viruses						
	SSII (2009)			SSIII (2010)			
	Myoviruses	Sipho-, Pleomorphic, icosahedral Viruses	Sipho-, Pleomorphic, Icosahedral Viruses (cc ^a^)	Myoviruses	Myoviruses (cc)	Sipho-, Pleomorphic, Icosahedral Viruses	Sipho-, Pleomorphic, Icosahedral Viruses (cc)
SSII	19 (3.8)^b^	9 (2.3)	2 (0.5)	42 (4.2)	66 (6.0)	-	2 (1.0)
SSIII	7 (1.4)	1 (0.3)	-	17 (1.7)	21 (1.9)	1 (1.0)	-
CC	9 (1.8)	1 (0.3)	2 (0.5)	30 (3.0)	35 (3.1)	-	4 (0.5)

**a**. CC, viruses isolated on culture collection strains; **b**. The number of interactions per virus is shown in brackets.

## 4. Discussion

Temporal isolation of haloviruses and their host organisms in one environment has not, to our knowledge, been performed in the past using a culture-dependent approach. In this survey, optimized production and purification protocols were developed for all the 36 obtained virus isolates. This study together with the spatial halovirus screening published in 2012 with its 45 isolated haloarchaeal viruses [22], almost tripled the number of known archaeal virus isolates to ~130. Most microbiological studies on hypersaline environments have been performed in aquatic environments [30]. We isolated cells and viruses from both liquid and solid samples. Interestingly, salt crystals were the richest source of microorganisms, as also previously observed [22] (Table 1 and Supplementary S1). This indicates that solid salt might contain more halophiles and their viruses than salt water. When the isolated viruses were cross-tested with the isolated 36 unique archaeal strains, altogether 268 specific virus-host interactions were observed indicating maintenance of infectivity over a one year time period.

Morphotype distribution of the viruses isolated here resembles the one obtained during the spatial survey [22], highlighting the abundance of haloarchaeal myoviruses (Figure 2). Interestingly, several of these myovirus isolates had astonishingly broad host ranges, especially virus HCTV-12 (No. 21) infecting altogether 14 strains from five different genera (Figure 3). Such broad host ranges have not been observed before for archaeal viruses. However, bacterial myoviruses are known to have complex tail structures with several different tail fibers allowing the recognition of a wide variety of host receptors [50]. In addition, the contractile myovirus tails encase a specific central tube structure which serves to penetrate the host cell envelope with greater physical force than the flexible sipho- and podovirus tails. Myoviruses have also been reported to exchange their host-specific genetic modules for receptor binding proteins and thus extend their host range and adaptation to different environments [51]. In this respect, the results obtained here suggest potential similarities among archaeal and bacterial myoviruses. Furthermore, it is possible that the observed extremely broad host ranges are characteristic for archaeal myoviruses. The other viral morphotypes in our study, siphoviruses, pleomorphic viruses, and especially the icosahedral inner membrane-containing virus, were rarer and more specific to a certain host. Opposing the broad host ranges of our myoviruses, high virus sensitivity was characteristic for many of our archaeal *Halorubrum* isolates although this feature was also observed to vary among the closely related strains (Figure 1 and Figure 4).

Several culture-independent studies have suggested that head-tailed viruses are scarce in hypersaline environments compared to, for example, the lemon-shaped virus-like particles [11,12,52]. Keeping in mind that only a small percentage of the natural strains are cultivable by the current methods, the strains and viruses obtained here represent a small subset of the microbiota in the Samut Sakhon saltern which does not necessarily portray the true diversity in the environment. The culture-dependent approach is biased to detecting only viruses that produce plaques on such hosts that grow as a proper lawn on artificial growth media. This does not, however, exclude the detection of non-lytic viruses as such viruses can be recognized by hazy plaques indicating host growth retardation. Moreover, the results of our previous spatial [22] and the current temporal culture-dependent studies (Figure 2) suggest that even if these viruses were scarce in the environment, they have a dynamic and persistent role, attacking archaeal cells over time and over the genus “barrier”. Interestingly, in a recent study of Lake Tyrrell, it was concluded that the most frequently detected CRISPR (clustered regularly interspaced palindromic repeats) [53] spacers targeted rare viruses, and such viruses were considered more stable during the 1–3 year periods than the most abundant ones [54].

A closer glance at the temporal virus-host interaction network of culturable strains and viruses of the Samut Sakhon saltern (Figure 4) reveals a few interesting trends. First, the overall distribution of strains in the two samples was different, which affects the number of obtained interactions, especially in the case of such viruses that are specific to certain hosts. Archaeal strains obtained during the year 2009 (SSII sample set) are favored by both, endogenous viruses, and viruses isolated a year after (SSIII sample set). SSIII viruses that were originally isolated on culture collection strains have the highest number of interactions (66) with SSII strains. This might imply that SSIII strains are more resistant to viruses isolated during the previous year, while SSII strains are more sensitive to the viruses obtained a year later. On the other hand, the high number of both endogenous and cross-sample interactions observed for SSII strains indicates that these strains are sensitive to viruses regardless of their isolation year. This might be partly explained by the high number of *Halorubrum* strains obtained from SSII samples, indicating that the presence of such strains might increase the number of obtained virus-host interactions. The phenomenon of many viruses attacking the culture collection strains, which originate from distant environments, supports the previous observations of global distribution of related microorganisms and their viruses in hypersaline environments [4,22,55,56,57,58].

Temporal fluctuations of viral populations studied by metagenomics in the hypersaline Lake Tyrell, Australia, represent the most extensive temporal culture-independent survey performed to date in hypersaline environments [3,28]. Halovirus populations were observed to be stable for a few days’ time periods, but dynamic when sampling intervals were extended until up to three years [3]. From the spatial point of view, more similarity was detected among viral assemblages within a certain sampling site as opposed to neighboring locations [28]. In addition, low number of hits was detected to previously described halovirus genomes. This is, however, expected taken in account the low number of sequenced halovirus genomes in public databases. One should also keep in mind that without the virus isolate and its GenBank sequence, reliable identification of true viral genomes is not possible. Extensive virus isolations and characterizations, such as this survey, are needed for more fundamental data mining of culture-dependent and -independent data sets. Nevertheless, studies on viral metagenomes can bring up different aspects of virus-host interactions than studies using virus and host isolates, and thus these two complementary approaches should go hand in hand instead of being directly compared to each other. The high maintenance of infectivity over a one-year period observed here supports the dynamic characteristics of halophilic microorganisms indicating a constant interplay between virus attacks and host resistance mechanisms. The low diversity of virus morphotypes obtained from the samples indicates that viruses with novel morphotypes are not commonly isolated although the true viral diversity in the samples is probably higher than observed here. The current numbers of described euryarchaeal and crenarchaeal virus morphotypes are six and 12, respectively [6]. Because the tailless icosahedral and short-tailed lemon-shaped virus morphotypes are shared by eury- and crenarchaeal viruses, the total number of archaeal virus morphotypes is 16. Even less morphotypes are known for bacteriophages [6]. Moreover, the low number of obtained virus morphotypes supports the structure-based viral lineage hypothesis that only a few protein folds are capable of forming an infectious virion [18,20,59,60]. More detailed information on virion structures is needed to determine the structural relationships, and the new viral isolates are a potentially valuable source for such information.

## Supplementary Files

Supplementary File 1
