# Supplementary material for: Archaeal Viruses Multiply: Temporal Screening in a Solar Saltern
Source: Viruses. 2015 Apr 10;7(4):1902–26. doi: 10.3390/v7041902 (PMC4411682; doi:10.3390/v7041902)
Supplement: Supplementary File 1 [file viruses-07-01902-s001.pdf]

Supplementary

Table S1. Samples used in the study.

Sample		Sampling Date	Source	Type	Density, g mL^{-1}
SSII	SS6	November 2009	Salt field *	Salt (fine grind)	
	SS7		Salt field *	Salt (large crystals)	
	SS8		Salt field **	Salt crystals	
	SS9		Saltern pond **	Salt water and mud	1.15
SSIII	SS10	December 2010	Salt field *	Salt (fine grind)	
	SS11		Saltern pond **	Salt water	1.02
	SS12		Saltern pond **	Salt water	1.05
	SS13		Salt field **	Salt crystals	

*Collected and rinsed by Samut Sakhon saltern personnel; **Collected by Academy Professor D. Bamford.

Table S2. Bacterial strains isolated and used in this study.

Sample [a]	Strain	16S rRNA Gene Partial Sequence GenBank Acc. No. and Length (bp)
SSII	*Rhodovibrio* sp. SS9-9	KM019181 (498)
SSIII	*Halobacillus* sp. SS10-1	KM019182 (515)
SSIII	*Halobacillus* sp. SS10-2	KM019183 (545)
SSIII	*Halobacillus* sp. SS10-8	KM019184 (545)
SSIII	*Pontibacillus* sp. SS11-1	KM019185 (872)
SSIII	*Halobacillus* sp. SS11-2	KM019186 (819)
SSIII	*Halobacillus* sp. SS11-4	KM019187 (966)
SSIII	*Halobacillus* sp. SS12-1	KM019188 (514)
SSIII	*Halobacillus* sp. SS12-2	KM019189 (526)
SSIII	*Halobacillus* sp. SS12-3	KM019190 (703)
SSIII	*Pontibacillus* sp. SS13-1	KM019191 (531)
SSIII	*Halobacillus* sp. SS13-2	KM019192 (556)
SSIII	*Pontibacillus* sp. SS13-3	KM019193 (506)
SSIII	*Sedimibacillus* sp. SS13-9	KM019194 (627)
SSIII	*Pontibacillus* sp. SS13-15	KM019195 (883)

a. SSII, Samut Sakhon sample 2009; SSIII, Samut Sakhon sample 2010.

Table S3. Host range of SSIII virus isolates with unknown morphology.

**Virus Isolate Number**	**37**	**38**	**39**	**40**	**41**	**42**	**43**	**44**	**45**
Initial stock titer, PFU ml^{-1}	1.8×10^8	1.3×10^6	2.9×10^7	1.5×10^8	1.6×10^7	1.7×10^7	6.2×10^9	1.2×10^6	1.7×10^6
Host strain									
Halorubrum sp. SS10-3							−2		
Halorubrum sp. SS10-9	1[a]	−1	1	1	1	1	1		−2
Haloarcula sp. SS13-14									H
Halorubrum sp. SS6-2	1	−1	1	1		1	1		1
Halobacterium sp. SS6-4									
Halobacterium sp. SS6-5	−1		−1		1	−2	−5		−2
Halorubrum sp. SS7-4	+1	−1	−2	−1	−2	−2	+1	−1	+2
Halorubrum sp. SS8-2	−1	−2		−2	1		−5	−4	+1
Haloarcula sp. SS8-4						−2			
Halorubrum sp. SS8-7	1	−1	1			1			
Halorubrum sp. SS9-12	−1	−2			1	1	−1	−1	
Halorubrum sp. SP3-3	−1	−2	1	1		1	−1		
Halorubrum sp. SS1-3	−1	−1	1	−2	1	1	1	−3	1
Har. japonica								H	
Hrr. sodomense	H	H	H	H	H	H	H		

a: The efficiency of plating (EOP) of the virus on its original host has been set to one and marked with H. Number 1 is equal to the EOP on the original host. Numbers −1, −2,… refer to 10^{-1}, 10^{-2},… and +1, +2,… refer to 10^{1}, 10^{2},… when compared to the EOP on the original host.

Table S4. Host range of myoviruses and siphoviruses.

**Morphotype**	**Myoviruses**																											**Siphoviruses**			
**Virus**	**HRTV-13**	**HRTV-14**	**HRTV-15**	**HRTV-16**	**HRTV-17**	**HSTV-4**	**HRTV-18**	**HRTV-19**	**HRTV-20**	**HRTV-21**	**HRTV-22**	**HRTV-23**	**HRTV-24**	**HRTV-25**	**HCTV-6**	**HCTV-7**	**HCTV-8**	**HCTV-9**	**HCTV-10**	**HCTV-11**	**HCTV-12**	**HCTV-13**	**HCTV-14**	**HCTV-15**	**HJTV-3**	**HRTV-26[b]**	**HRTV-27[b]**	**HRTV-28**	**HRTV-29**	**HATV-3**	**HCTV-16**
**Virus number**	**1**	**2**	**3**	**4**	**5**	**6**	**7**	**8**	**9**	**10**	**11**	**12**	**13**	**14**	**15**	**16**	**17**	**18**	**19**	**20**	**21**	**22**	**23**	**24**	**25**	**26**	**27**	**28**	**29**	**30**	**31**
Host strain																															
Halorubrum sp. SS10-3	−2[a]	-5				1	H	H	1			-3				-7		-7	-7	-7	-7		-8								
Halorubrum sp. SS10-9	−1	1	+1		1	1	−1	−3	H	H	H	H	H	1	−1	1	−1	−1	−1	−1	−1	−1	−6	−2		−1	1	−10			
Halorubrum sp. SS13-12														H																	
Halorubrum sp. SS6-1		−6							−6						−4				−7		−5										
Halorubrum sp. SS6-2	−1	H	H	H		1	1	+1	1	1		−1	1	−1	−1	−3	−4	−4	−4	−3	−3	1	−3	1	−6	−1					
Halorubrum sp. SS7−4		−2				1	1	−6	−1				−2		−3								−8	−8				−5	H	−4	−7
Halorubrum sp. SS8-2	H	−1	−7	−6		−1	−1	1	−1	−1	1	1	1		−1	+1	+1	+1	1	1	1	−1	1		−5	−4		−10			
Halorubrum sp. SS8-7						−4	−4	−6	−2	1				−8	−1	1	1	1	1	−2	1	−2	−1	−5		−1		H			
Halorubrum sp. SS9-12	−1	1	−6		H	1	−1	1	−1	−1	−1	−1	−1	−1			−7	−7		−7	−7		−8	−8	−7	−1					
Halorubrum sp. SS5-4						−1	−1	1	1			+1															−1				
Halorubrum sp. SP3-3	−2	−3			−3	+1	−1	−3	−1	1	−7	−1	−4												−6	−1					
Halorubrum sp. SS1-3	1	−5	−1	−2		1	1	1	1	1	1	1	1		1	−1	1	1	1	1	1	−1	−1	1	1	−1	1				
Halorubrum sodomense		−1		−6		H			−6	1	−1		−1		1	1	+1	+1	1	1	1	1	1	1		−1					
Haloterrigena sp. SS13-7				1															−3	−1	1										
Halobacterium sp. SS6-4	−1				1											1	1	1	−1	1	1		−1					−10			
Halobacterium sp. SS6-5						−5			−5							1				−8	−8		−1								
Halobellus sp. SS6-7																−1	−1			−1	−1		−1								
Haloarcula sp. SS8-4	−2	−1			−1	−1	−3								−1							−1			1						
Haloarcula sp. SS8-5																												−5		H	
”Haloarcula californiae”										−4					H	H	H	H	H	H	H	H	H	H							H
Haloarcula japonica																									H						
Haloarcula hispanica																														−5	

a: The efficiency of plating (EOP) of the virus on its original isolation host has been set to one and marked with H. Number 1 is equal to the EOP on the original host. Numbers −1, −2,… refer to 10^{-1}, 10^{-2},… and +1, +2,… refer to 10^{1}, 10^{2},… when compared to the EOP on the original host; **b**: The host for viruses HRTV-26 and HRTV-27 is *Halorubrum* sp. SS13-13, which occasionally did not form a lawn, and consequently, it was left out from the cross-titration. The titers of HRTV-26 and HRTV-27 on *Halorubrum* sp. SS13-13 were 2×10^{8} and 5×10^{6} PFU ml^{-1}, respectively, which were marked here as one and used here for comparison.

Table S5. Host range of pleomorphic and icosahedral viruses.

	**HRPV-6**	**HRPV-7**	**HRPV-8**	**HAPV-1**	**HCIV-1**
**Virus number**	**32**	**33**	**34**	**35**	**36**
Host strain					
Halorubrum sp. SS7-4	H	1 [a]			−6
Halorubrum sp. SS8-2			−1		
Halorubrum sp. SS5-4		H			
Halorubrum sp. SP3-3			H		
Haloarcula sp. SS13-14				H	
”Haloarcula californiae”					H
Haloarcula japonica					1
Haloarcula hispanica					−6

a. The efficiency of plating (EOP) of the virus on its original host has been set to one and marked with H. Number 1 is equal to the EOP on the original host. Numbers −1, −2,… refer to 10^{-1}, 10^{-2},… and +1, +2,… refer to 10^{1}, 10^{2},… when compared to the EOP on the original host.

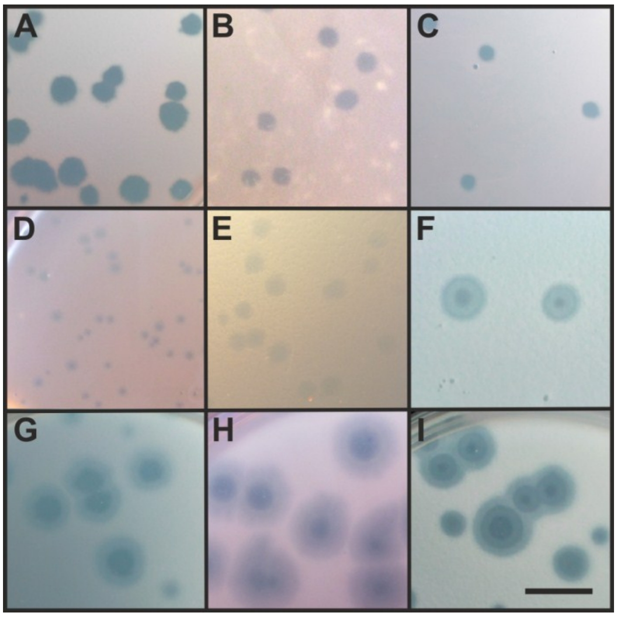

Figure S1. Different plaque morphologies. (**A**–**C**) Clear plaques; (**D**–**I**) turbid plaques. (**A**) Myovirus HRTV-22; (**B**) icosahedral virus HCIV-1; (**C**) myovirus HRTV-18; (**D**) siphovirus HRTV-29; (**E**) isolate No 37 with unknown morphotype; (**F**) myovirus HCTV-10; (**G**) myovirus HCTV-9; (**H**) pleomorphic virus HRPV-6; (**I**) myovirus HCTV-11. Scale bar, 1 cm for all sections.

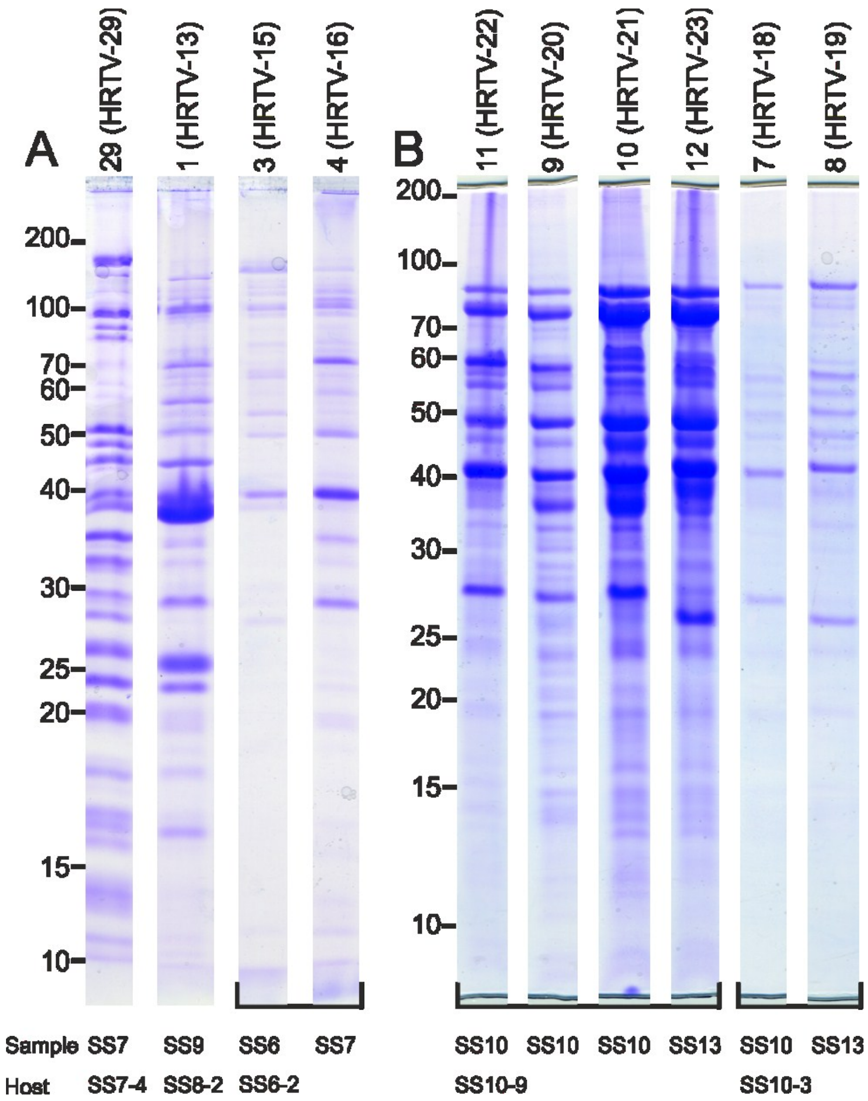

Figure S2. Structural protein profiles of the purified virus isolates analyzed in Coomassie Brilliant Blue stained tricine-SDS-polyacrylasmid gel. Virus numbers refer to Table 1. (**A**) Examples of virus isolates with different protein profiles. Virus No. 29 is a siphovirus, Nos. 1, 3, and 4 are myoviruses; (**B**) Examples of myovirus isolates with closely related protein profiles. The molecular mass marker (kDa) is shown on the left in A and B. The origin (sample; see Supplementary Table S1) and the isolation of hosts (all belong to *Halorubrum*) of the viruses are shown below.
